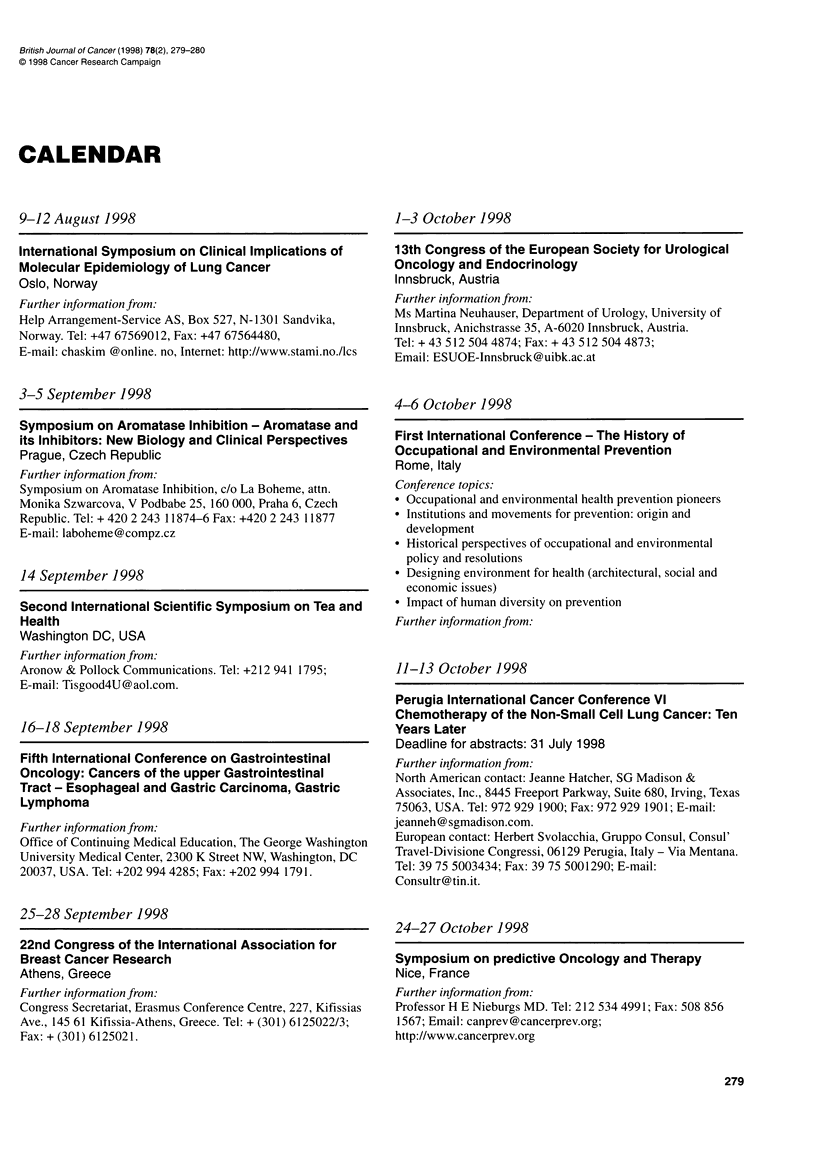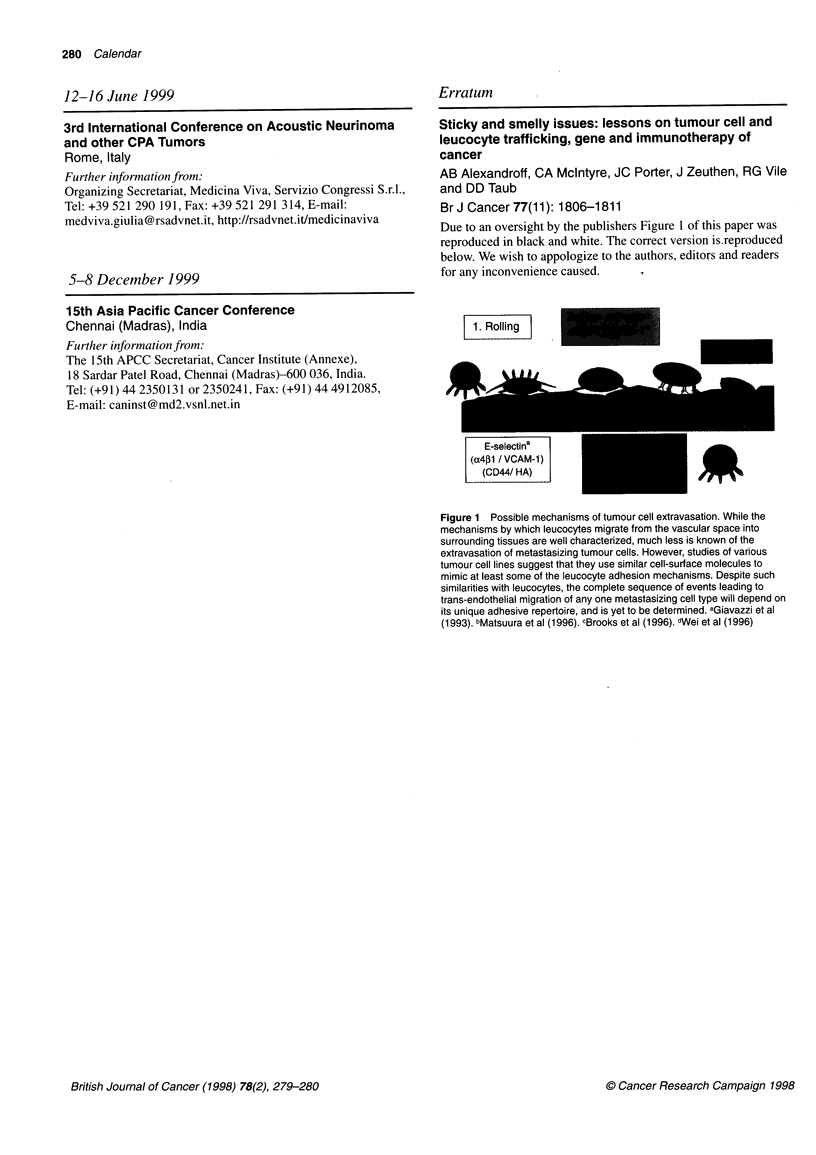# Calendar

**Published:** 1998-07

**Authors:** 

## Abstract

**Images:**


British Journal of Cancer (1998) 78(2), 279–280
© 1998 Cancer Research Campaign

### CALENDAR

### *9–12 August 1998*

**International Symposium on Clinical Implications of Molecular Epidemiology of Lung Cancer**
Oslo, Norway

*Further information from:*
Help Arrangement-Service AS, Box 527, N-1301 Sandvika, Norway. Tel: +47 67569012, Fax: +47 67564480, E-mail: chaskim @online. no, Internet: http://www.stami.no./lcs

### *3–5 September 1998*

**Symposium on Aromatase Inhibition – Aromatase and its Inhibitors: New Biology and Clinical Perspectives**
Prague, Czech Republic

*Further information from:*
Symposium on Aromatase Inhibition, c/o La Boheme, attn. Monika Szwarcova, V Podbabe 25, 160 000, Praha 6, Czech Republic. Tel: + 420 2 243 11874–6 Fax: +420 2 243 11877 E-mail: laboheme@compz.cz

### *14 September 1998*

**Second International Scientific Symposium on Tea and Health**
Washington DC, USA

*Further information from:*
Aronow & Pollock Communications. Tel: +212 941 1795; E-mail: Tisgood4U@aol.com.

### *16–18 September 1998*

**Fifth International Conference on Gastrointestinal Oncology: Cancers of the upper Gastrointestinal Tract – Esophageal and Gastric Carcinoma, Gastric Lymphoma**

*Further information from:*
Office of Continuing Medical Education, The George Washington University Medical Center, 2300 K Street NW, Washington, DC 20037, USA. Tel: +202 994 4285; Fax: +202 994 1791.

### *25–28 September 1998*

**22nd Congress of the International Association for Breast Cancer Research**
Athens, Greece

*Further information from:*
Congress Secretariat, Erasmus Conference Centre, 227, Kifissias Ave., 145 61 Kifissia-Athens, Greece. Tel: + (301) 6125022/3; Fax: + (301) 6125021.

### *1–3 October 1998*

**13th Congress of the European Society for Urological Oncology and Endocrinology**
Innsbruck, Austria

*Further information from:*
Ms Martina Neuhauser, Department of Urology, University of Innsbruck, Anichstrasse 35, A-6020 Innsbruck, Austria. Tel: + 43 512 504 4874; Fax: + 43 512 504 4873; Email: ESUOE-Innsbruck@uibk.ac.at

### *4–6 October 1998*

**First International Conference – The History of Occupational and Environmental Prevention**
Rome, Italy

*Conference topics:*
- Occupational and environmental health prevention pioneers
- Institutions and movements for prevention: origin and development
- Historical perspectives of occupational and environmental policy and resolutions
- Designing environment for health (architectural, social and economic issues)
- Impact of human diversity on prevention

*Further information from:*

### *11–13 October 1998*

**Perugia International Cancer Conference VI Chemotherapy of the Non-Small Cell Lung Cancer: Ten Years Later**
Deadline for abstracts: 31 July 1998

*Further information from:*
North American contact: Jeanne Hatcher, SG Madison & Associates, Inc., 8445 Freeport Parkway, Suite 680, Irving, Texas 75063, USA. Tel: 972 929 1900; Fax: 972 929 1901; E-mail: jeanneh@sgmadison.com.
European contact: Herbert Svolacchia, Gruppo Consul, Consul' Travel-Divisione Congressi, 06129 Perugia, Italy – Via Mentana. Tel: 39 75 5003434; Fax: 39 75 5001290; E-mail: Consultr@tin.it.

### *24–27 October 1998*

**Symposium on predictive Oncology and Therapy**
Nice, France

*Further information from:*
Professor H E Nieburgs MD. Tel: 212 534 4991; Fax: 508 856 1567; Email: canprev@cancerprev.org; http://www.cancerprev.org

### *12–16 June 1999*

### 3rd International Conference on Acoustic Neurinoma and other CPA Tumors
Rome, Italy

*Further information from:*
Organizing Secretariat, Medicina Viva, Servizio Congressi S.r.l., Tel: +39 521 290 191, Fax: +39 521 291 314, E-mail: medviva.giulia@rsadvnet.it, http://rsadvnet.it/medicinaviva

### *5–8 December 1999*

### 15th Asia Pacific Cancer Conference
Chennai (Madras), India

*Further information from:*
The 15th APCC Secretariat, Cancer Institute (Annexe), 18 Sardar Patel Road, Chennai (Madras)–600 036, India. Tel: (+91) 44 2350131 or 2350241, Fax: (+91) 44 4912085, E-mail: caninst@md2.vsnl.net.in